# Memory Impedance in TiO_2_ based Metal-Insulator-Metal Devices

**DOI:** 10.1038/srep04522

**Published:** 2014-03-31

**Authors:** Li Qingjiang, Ali Khiat, Iulia Salaoru, Christos Papavassiliou, Xu Hui, Themistoklis Prodromakis

**Affiliations:** 1 College of Electronic Science and Engineering, National University of Defense Technology, Changsha 410073, P. R. China; 2 Nano Research Group, School of Electronics and Computer Science, University of Southampton, Southampton SO17 1BJ, UK; 3 Department of Electrical and Electronic Engineering, Imperial College London, London SW7 2AZ, UK

## Abstract

Large attention has recently been given to a novel technology named memristor, for having the potential of becoming the new electronic device standard. Yet, its manifestation as the fourth missing element is rather controversial among scientists. Here we demonstrate that TiO_2_-based metal-insulator-metal devices are more than just a memory-resistor. They possess resistive, capacitive and inductive components that can concurrently be programmed; essentially exhibiting a convolution of memristive, memcapacitive and meminductive effects. We show how non-zero crossing current-voltage hysteresis loops can appear and we experimentally demonstrate their frequency response as memcapacitive and meminductive effects become dominant.

Classical circuit theory is founded on the axiomatic definition of three fundamental circuit elements: the resistor by Ohm^1^, the inductor by Faraday^2^, and the capacitor by Volta^3^. These definitions however are only static descriptions of the instantaneous values of the corresponding variables, despite the fact that dynamic responses have been observed well before the establishment of these definitions^4^. About forty years ago, Chua envisioned the concept of memory-resistors (memristors) based-upon a symmetry argument^5^ that imposed the existence of a missing fundamental circuit element that provided linkage between charge and flux (Figure S1a). Whilst this argument is considered to be fair, the later generalised definition by Chua and Kang^6^ is of more fundamental importance as it provides a state-dependent relationship between current and voltage that broadly captures dynamic resistive elements^7^. Originally the broad generalization of “memristors” was conceived as a new class of dynamical systems and as such it was referred as “memristive systems”. To avoid confusion, we follow the latest notation imposed by Chua, the lead author of both reports, and we refer to this class of devices as “memristors”. This is in tandem with the denomination of capacitors and inductors; practical implementations are not explicit facsimile to the corresponding ideal definitions yet these are not called capacitive or inductive systems.

Similarly, two new auxiliary classes of dynamical elements were coined up^8^ as memory-capacitors (memcapacitors) and memory-inductors (meminductors) that establish state-dependent relationships among charge-voltage (Figure S1b) and current-flux (Figure S1c) respectively. The common property of these distinct subsets is memory, which is attributed to inertia between the causal stimulus and the diverse range of physical mechanisms that support the various state modalities. The signature of this inertia is a pinched hysteresis loop in the i-v, q-v and ϕ-i domain respectively for memristors, memcapacitors and meminductors (Figures S1d,e,f); notwithstanding the classical definitions of resistors, capacitors and inductors that are described by single-valued functions and should thus be considered as special cases of these broad subsets (Figure 1a).

To date, the research community has shown great interest on demonstrating exclusive solid-state implementations of memristors^9^,^10^,^11^, memcapacitors^12^,^13^,^14^ and meminductors^15^,^16^; some of which are highlighted in a review by Pershin and Di Ventra^17^. Deliberate attempts to fabricate practical cells, past HP's work on memristors^11^ and the supplementary theoretical definitions for memcapacitors and meminductors^8^, have strived to match the characteristics dictated by the ideal definitions, while reports on related phenomena that were incidentally observed were more relaxed. There is serious contention among scientists particularly for memristors, the most exploited subset of devices so far is the non-zero crossing i-v characteristics of solid-state implementations which is considered to be contradicting the original theoretical conception^18^, arguing the need for revamping the existing memristor theory; a plausible extension is to incorporate a nano-battery effect^19^.

In our opinion, the global definition of memristors^6^ is well-defined. Such phenomena simply manifest the co-existence of distinct memory modalities^20^, which collectively facilitate a response that inevitably differs from the classical theoretical definition; one should be able to disentangle the individual contributions within single devices. The coexistence of parasitic effects has been observed on practical devices for more than a century^21^,^22^, with this effect being more apparent when the devices' characteristics are exploited in broad frequency spectrums where the static parasitic contributions are more notable. We argue that practical solid-state devices would experience dynamic parasitic contributions and it is thus more appropriate to investigate the devices' response utterly as memory impedance. Figure 1a demonstrates conceptually how such a complex interaction emerges by the mingling of distinct memory effects, namely memristive, memcapacitive and meminductive. Particularly in case **I** (**III**) the corresponding i-v crossing point would occur within the first (third) quadrant as illustrated in Figure 1b (1c), due to an additive memcapacitive (meminductive) contribution. In case **IV** however, all three fundamental memory effects are expressed in a device and the i-v crossing point could either occur within the first or third quadrant as shown in Figure 1d, depending upon the device's impedance constituent memory elements (Figure 1d inset), whose dominance is determined via the stimulus frequency.

## Non-zero-crossing behaviour of TiO_2_ based metal-insulator-metal devices

Here, we experimentally demonstrate the coexistence of dynamic parasitic effects in the prototypical Metal-Insulator-Metal (MIM) single crossbar architecture based on a TiO_2+x_/TiO_2_ functional core^23^; one of our prototypes is illustrated in the inset of Figure 2a. Multiple devices were fabricated, with the fabrication flowchart outlined in the Methodology section. For all devices, we initially employ a quasi-static ±3 V voltage sweep for acquiring the characteristic pinched-hysteresis i-v loop that is the well-known memristor signature^24^. Figure 2a shows the i-v signature of a Pt/TiO_2+x_/TiO_2_/Pt cell with an active area of 5 × 5 μm^2^. In this case, we observed that the crossing point occurs at 1 V, indicating the presence of some parasitic capacitance. The influence on this parasitic capacitance was explored while programming the device at bipolar states (see Figures S2a, b for programming/evaluating procedure). Figures 2b and 2c demonstrate the concurrent resistive/capacitive switching of our prototype, toggling from a high-resistive state (HRS) to a low-resistive state (LRS) and correspondingly from a high-capacitive state (HCS) to a low-capacitive state (LCS). These experimental results confirm that the memristive behavior is indeed accompanied by a memcapacitive behavior. It is worthy to point out that the capacitance switching ratio is frequency dependent^25^. As demonstrated in Figure 2c, the difference between HCS and LCS is significantly larger at the lower C-V test frequency (100 KHz). Unless otherwise stated, all C-V tests were implemented at 1 MHz, a frequency that is near to the RC pole of the device. Thus, the capacitive switching ratio is lower than that of resistance extrapolated from DC pulses. Similar experimental results have been previously demonstrated in other ReRAM devices^20^,^25^. In this manuscript, all tested devices were electrically characterized without employing an electroforming step. As a result, the activation energy supplied by a single set or reset pulse is not sufficient to generate formation and rupture of continuous filaments. Resistive switching events are thus not available at each programming pulse, rather at multiple pulses that facilitate a collective behavior, as demonstrated in Figures 2b and 2c.

In this particular case, the memristive/memcapacitive switching are correlated; a phenomenon that has previously been observed in perovskite^25^. It is worth mentioning though that the coexistence of memristive and memcapacitive behaviors have also been observed on other MIM cells from the same wafer with 2 × 2 μm^2^ and 10 × 10 μm^2^ active areas. Interestingly, the programming memristance and memcapacitance of devices of 2 × 2 μm^2^ active area are anti-correlated, as shown in Figure S2c, while devices with larger active areas, i.e. 5 × 5 μm^2^ and 10 × 10 μm^2^, follow alike switching trends. Similar opposing memristor – memcapacitor programming trends have been reported recently^26^, with the programming and evaluation of the two memory properties though being executed independently one from another. The reason for the area dependence of the relationship between capacitance and resistance is argued to be due to distinct dominant conducting mechanisms. It should be noted that the measured data in devices with active area of 2 × 2 μm^2^ was attained under bipolar switching mode, where schottky barriers at both the top and bottom interfaces are anticipated to play a dominant role. In this case, positive programming pulses could decrease the top interface resistance and its related capacitance, but it would not necessarily increase the bottom interface resistance and its related capacitance due to the pulse's saturation limit^25^,^27^. As a result, the device's effective resistance will decrease due to the shunting of the top space charge region. In turn, the total capacitance of the whole device will increase because its value is now dominated mainly by the bottom interface^25^. Similar explanations apply for negative programming pulses. To further explore any possible influence of the device's electrodes on the measured capacitance, we implemented C-V tests on pristine devices of varying electrode areas (2 × 2 μm^2^, 5 × 5 μm^2^, and 10 × 10 μm^2^). As expected, the measured initial capacitance is in proportion to the electrode areas and the capacitance per unit electrode area is ascertained to be C_unit_ = 18 (fF/μm^2^). Detailed arguments could be found in Supplementary materials.

The experimental results shown in Figure S3 in the supplementary materials serve as evidence that the functional mechanism of resistance modulation in our MIM devices is indeed filamentary in nature. We thus argue that for the device to assume a LRS, the TiO_2_ core will locally undergo substantial reduction (TiO_2-x_) that will support one or multiple continuous current percolation filaments that would effortlessly conduct current from the top (TE) to the bottom electrode (BE), as demonstrated in Figure 2d. It should be noted that the filament in TiO_2_ based resistive devices has a bulky conical shape, which has been experimentally demonstrated in recent studies^28^,^29^. In the case however that the device is programmed at HRS, this filament would be annihilated (or partially formed), resulting in a barrier region (L_1_) among any existing percolation branch and the BE. Yu et al.^30^ pointed out that such a barrier would render a poor DC conduction that can be modelled as a capacitance, nonetheless they overlooked the fact that this is essentially an auxiliary memcapacitance, as theoretically denoted by Mouttet^31^. Depending on the polarity of the applied potentials, this barrier would decrease or increase that in turn would set the corresponding static resistive and capacitive states. While the device is programmed at a HRS, measured results acquired by impedance spectroscopy would denote that the device could be statically modelled as a parallel combination of a resistor (R_1_) and a capacitor (C) in series with a small resistor (R_0_), as shown in Figure 2e. R_0_ represents all other Ohmic resistances including that of the initial conducting filaments and measurement connections, which is usually no more than 20 Ω overall^32^. One would argue that when a continuous filament is formed, resulting into LRS, this barrier would diminish, rendering minimum static values for both resistance and capacitance. This is indeed illustrated in Figure 2f, where the measured results on the presented Nyquist plot cluster together on the Re (Z) axis.

A series of repeated impedance measurement cycles was implemented on the 10 × 10 μm^2^ active cell with the acquired results shown in Figures 3a–3b (the corresponding programming- evaluating procedures are shown in Figures S2a, b). The 10 × 10 μm^2^ cells demonstrate a rather interesting response, as shown in Figure 3b, that directly contradicts the unipolar impedance measurements acquired from 5 × 5 μm^2^ active cells. In this case, the applied stimuli cause the device's reactance to toggle between both negative and positive values, indicating that capacitive and inductive behaviours are alternately dominating. The measured reactance values in this case are treated as the ‘net' contribution of a concurrent capacitive and inductive response. Our measured results thus indicate that a functional-oxide based MIM capacitor of relatively large active area (in this case ≥ 10 × 10 μm^2^) can effectively support concurrently all three memory states. The origin of this triple-state coexistence and the distinct programming trends can possibly be explained via the filamentary formation/annihilation that occurs within the active core of our prototypes due to a redox mechanism of TiO_2_. In HRS, large resistive states would ascertain large tunnelling gaps (L_1_) between the device's electrodes, and thus should introduce a significant capacitive effect. A larger active area, as in the case of our 10 × 10 μm^2^ prototype, in principle allows percolation channels to occur over larger volumes, essentially facilitating the formation of winding conductive paths that will inevitably introduce a notable effective inductance. But in HRS, the filaments density and path tortuosity are limited, and thus the capacitive effects are dominant. In contrast, the CF is fully shorting TE and BE in LRS, which would minimise the capacitive effect, while introduce the dominant inductive effect from a number of winding filaments as shown in Figure 3d. This is also verified in Figures S2c–S2e, which clearly demonstrated that the conductivity of LRS is correlated with the cell size.

## Frequency response of *i*-*v* characteristics

**Figure 1:**
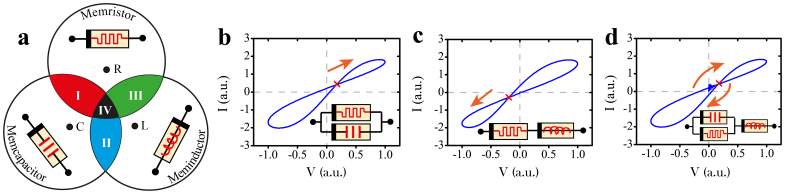
Classification of Memory Impedance. (a) Illustration of the three fundamental subsets of dynamic elements, with the overlapping areas representing plausible complex memory expressions in practical devices. Non-zero crossing i-v characteristics are attributed to the coexistence of distinct memory features; shown are distinct cases of (b) memristance and memcapacitance coexistence, (c) memristance and meminductance coexistence and (d) coexistence of all memory attributes in a single device. It should be noted that the orange arrows in (b)–(d) indicate the direction of the crossing points.

**Figure 2:**
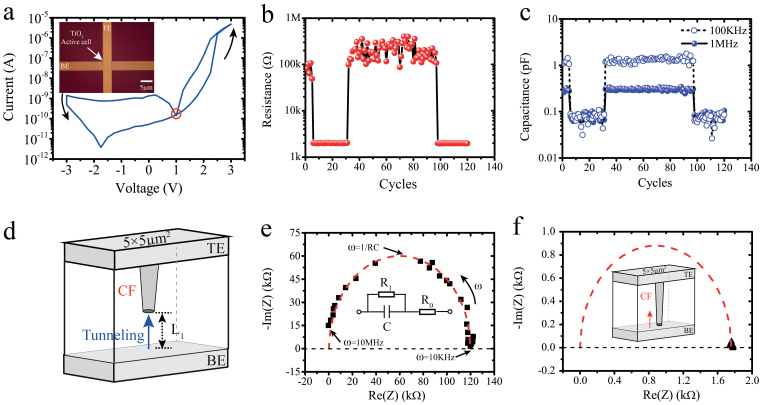
Measured features of practical TiO_2_-based MIM devices. (a) Measured i-v characteristics showing a non-zero-crossing behaviour. Inset: Optical microscope image of a prototype cell with an active area of 5 × 5 μm^2^. (b) Resistance and (c) capacitance programming occurs concurrently after pulsing the device. The capacitance values shown by solid cycles were measured at 1 MHz, while dash cycles represent results at 100 KHz. (d) Schematic of DC conduction processes inside a 5 × 5 μm^2^ active cell in HRS. As the ruptured distance L_1_ is relatively large, the tunnelling from the bottom electrode to the ruptured CF would block the DC conduction processes^25^, which would introduce larger capacitive effects. (e) Impedance spectrum of a 5 × 5 μm^2^ active cell in HRS, accomplished via a 10 mV AC signal (10 KHz–10 MHz, DC biasing point is 0 V). Inset: schematic of the equivalent circuit. The semicircle suggests the active cell could be modelled as a parallel of a resistor and a capacitor. (f) Impedance spectrum of the same active cell in LRS (1.8 KΩ). Inset: Schematic of DC conduction processes in LRS. After programming, a continuous CF is formed between TE and BE.

**Figure 3:**
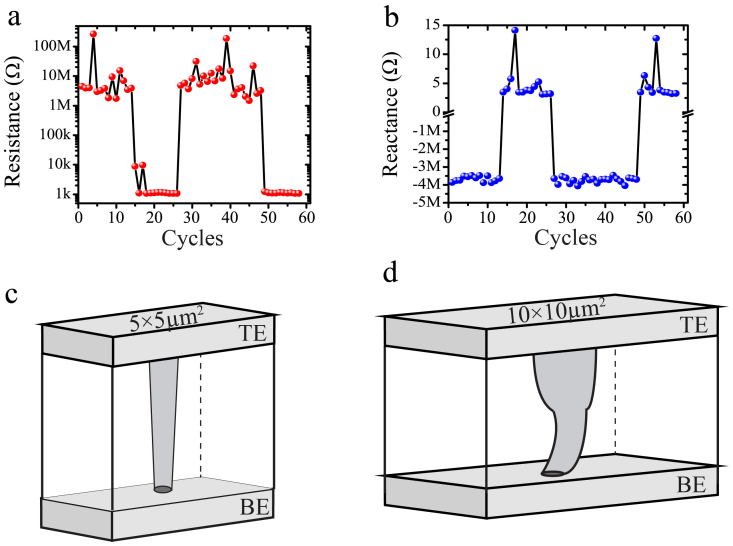
Impedance Features of a ReRAM cell with active area of 10 × 10 μm^2^. (a) Resistance changes in repeat measurement cycles at room temperature and (b) corresponding reactance trend, evaluated at 1 MHz. Schematic illustrations of conceptual conducting filaments, formed in a (c) 5 × 5 μm^2^ and (d) 10 × 10 μm^2^ active cells. The larger the active layer's volume, the higher the probability of observing an inductive percolating channel due to reduced TiO_2_ areas forming windings.

So far, all non-zero-crossing behaviours have been observed by employing sweeping potentials^19^ of static frequency. In order to prove our hypothesis that ReRAM cells concurrently support memristive, memcapacitive and meminductive components, we employed a sinusoidal stimulus of fixed voltage range at distinct testing frequencies. This approach allowed us to evaluate the effect on the i-v characteristics of the device under test and the shifting of the crossing point due to the dominance of the distinct memory components at distinct frequencies, as illustrated in Figures 1b, c, d. To ensure that we do not encounter any erroneous parasitic effects while evaluating the characteristics of single devices, we optimised our instrumentation setup and limited the measuring frequency spectrum up to 1 MHz. This setup was also benchmarked while measuring known SMD (Surface-mount device) components (Figure S4) that up to 1 MHz showed no significant parasitic effects. It should also be noted that to preserve devices from any hard-breakdown as well as minimising the effect of any switching thresholds asymmetry, at any single frequency point, only one sinusoidal period was applied from a Tektronix arbitrary function generator (AFG-3102).

**Figure 4:**
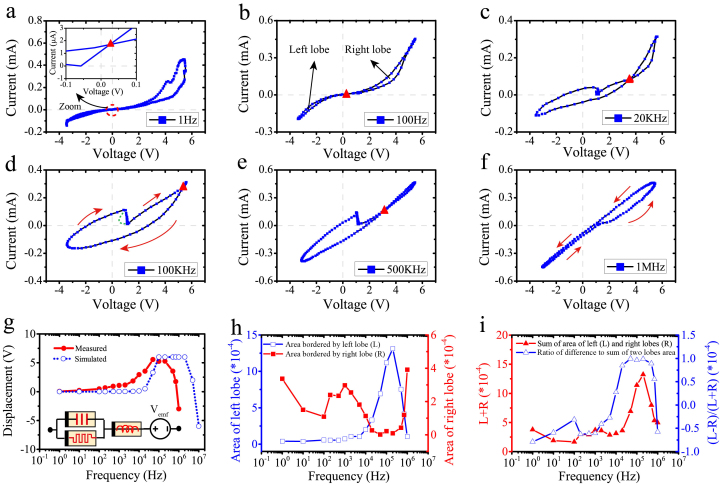
Frequency Responses of pinched hysteresis loops for packaged diced dies. The schematics of packaged diced dies and measured method are illustrated in Fig. S4a and b. (a)–(f) Measured i-v curves of the same device measured at frequencies from 1 Hz to 1 MHz. It should be noted that the green dashed line in (d) depicts a plausible form of i-v characteristic that can give rise to the observed step in (d) and (e). (g) Measured and simulated displacement of crossing points with increasing frequencies; inset depicts an equivalent circuit model (all model parameters can be found in Table S1). (h) Measured areas bordered by left (L) and right (R) lobes of the frequency-dependent i-v characteristics. (i) Sum and normalised difference of the hysteresis exhibited in the i-v characteristics at distinct frequencies.

Figure 4 depicts measured i-v characteristics of our 10 × 10 μm^2^ prototypes as the stimulus frequency ranged from 1 Hz to 1 MHz. Specifically, in the inset of Figure 4a, a small offset (40 mV) is observed at f = 1 Hz indicating the existence of a nanobattery^19^ (V_emf_), as the influence of parasitic effects could be neglected at such low frequency. By increasing the stimulus frequency to 100 Hz, we observe the shrinking of the area encountered within the right i-v lobe along with a slight increase in the crossing point offset, respectively captured in Figures 4g and 4h. The influence of a memcapacitive response starts to appear in Figure 4c, as the stimulus frequency is further increased at *f* = 20 KHz. The crossing i-v point is now clearly displaced to the first quadrant, while the hysteresis loop opens up from a lissajous towards a circular form, a characteristic of memcapacitance (Figure S1g) that becomes even more apparent at *f* = 100 KHz (Figure 4d). Interestingly, further increasing the stimulus frequency causes the crossing i-v point to drift towards the third quadrant, as predicted theoretically (Figure 1d) and shown here experimentally in Figures 4e and 4f. It is worthy to point out that there is a step in the i-v curves in Figures 4d and 4e. We argue this behaviour being similar to the steps observed on the i-v characteristics of some classes of diodes that include high internal field regions, and are subject to local breakdown, such as tunnel diodes and silicon controlled rectifiers. In the vicinity of the breakdown such devices can exhibit a local negative differential resistance without violating passivity. When the i-v characteristics of this class of devices are traced in conductance mode (source v from a small source impedance, measure i) or in impedance mode (source i from a small source admittance, measure v) without any special precaution to overcompensate the device's negative resistance, steps could be observed as illustrated in Figures 4d and 4e. A plausible form of i-v characteristic that can give rise to the observed step has been annotated in green on Figure 4d.

Overall, the exhibited hysteresis of our prototypes is large for low frequencies where the memristive component is dominant, and as frequency increases it reduces (as anticipated by the memristor theory), increases (as memcapacitive effects come into play) and then again reduces; as depicted in Figure 4i. At the same time, the crossing i-v point origins at 0 V denotes an almost purely passive component at low frequencies, with capacitive (inductive) components adding a positive (negative) offset at frequencies where the corresponding effects are more dominant as depicted in Figure 4g. Our experimental results were closely fitted with an equivalent circuit model (inset of Figure 4g) comprising a parallel combination of a memristor and memcapacitor that are serially connected to a meminductor and a nanobattery (detailed simulated methods and parameters can be found in Supplementary Materials). The areas bordered by the pinched hysteresis i-v loops were calculated as reported previously^33^,^34^. Figure 4h depicts the changes of left and right lobes' areas. It is clear that at frequencies below 100 Hz, the memristive effect was dominant, thus area of right lobes dropped; Considering the migration of crossing point to the first quadrant, the area of left lobe kept almost the same. Then at frequencies between 100 Hz to 200 KHz, memcapacitive effect was dominant, area of left lobes thus increased sharply; In case of right lobes, the area increased initially (100 Hz–1 KHz), but then went down as a result of the crossing point migration. Finally, meminductive effect became dominant above 200 KHz and the crossing point started to drift oppositely. Thus, the area of right lobes grew up whilst an opposite trend occurred for the left lobes. Figure 4i depicts the sum and normalised difference of the lobes' areas at distinct frequencies. It can be observed that at frequencies below 10 KHz, the right lobe hysteresis outweighs the left one. In contrast, an opposite ratio polarity could be obtained when memcapacitive effect is dominant (10 KHz to 700 KHz), while meminductive would invert the polarity again at even higher frequencies (above 1 MHz).

## Summary

In this work we presented experimental evidence that TiO_2_-based MIM devices, commonly known as memristors, exhibit concurrently memristance, memcapacitance and meminductance. We showed that these components are concurrently modulated under voltage biasing and we have identified that meminductance is more apparent for devices of large active areas. We also demonstrated that the frequency response of the devices' pinched-hysteresis i-v does not follow the classical signature of memristors, and it is a manifestation of all three memory components. We believe that these features can be particularly useful in developing adaptive circuits that operate in radio-frequencies^35^, while they open up the possibility of establishing self-resonating nanoscale components that could find applications in cellular neural networks and neuromorphic implementations^36^.

## Methods summary

### Fabrication of TiO_2_ based active cells

In this process flow, we used thermal oxidation to grow 200 nm thick SiO_2_ on 4″ silicon wafer and we employed optical lithography method to pattern the Bottom Electrodes (BE), Top Electrodes (TE) and the TiO_2_ active material. E-beam evaporation was employed to deposit 5 nm adhesive layer and 30 nm Pt layer as BE and 30 nm thick Pt as TE, followed by lift-off process to define the patterns. For TiO_2_, sputtering at 300 Watts and 2 min wet etching, in 1:50/HF: H_2_O solution, were performed. The design allows having Pt/TiO_2+x_/TiO_2_/Pt ReRAM structures in cross-bars and standalone configurations.

### Electrical measurements

Electrical measurements for active cells on wafer were performed utilising a low-noise Keithley 4200 semiconductor characteristics system combined with a probe station (Wentworth AVT 702). The i-v characteristics were firstly obtained via sweeping voltages, following distinct sequences for bipolar and unipolar resistive switching. Then, impedance spectroscopies were tested biasing a small 10 mV AC signal (DC bias is 0 V) with frequencies sweeping from 10 KHz to 10 MHz. To measure the changing trends of impedance, a series programming (5 V for SET, and −5 V for RESET) pulses were applied across active cells with a followed 0.5 V pulse to read resistance values. In all measurements, pulse widths were set to 10 μs. All reactance measurements were implemented via C-V tests by employing 30 mV 1 MHz AC signals (DC bias is 0.5 V). Specifically, the measuring option for devices with active areas of 2 × 2 μm^2^ and 5 × 5 μm^2^ was set to the parallel capacitance and conductance (C_p_-G_p_), while for devices with active area of 10 × 10 μm^2^, the measuring option was set to complex impedance (Z-Theta).

## Author Contributions

L.Q., C.P., I.S., A.K., T.P. and X.H. conceived the experiments. A.K. and T.P. fabricated the samples. L.Q. and I.S. performed the electrical characterization of the samples. L.Q. performed all simulations. All authors contributed in the analysis of the results and in writing the manuscript.

## Supplementary Material

Supplementary Information Memory Impedance in TiO_2_ based Metal-Insulator-Metal Devices
