# Supplementary material for: Memory Impedance in TiO2 based Metal-Insulator-Metal Devices
Source: Sci Rep. 2014 Mar 31;4:4522. doi: 10.1038/srep04522 (PMC3970129; doi:10.1038/srep04522)
Supplement: Supplementary Information — Memory Impedance in TiO2 based Metal-Insulator-Metal Devices [file srep04522-s1.pdf]

Supplementary Information

Memory Impedance in TiO_2 based Metal-Insulator-Metal Devices

Li Qingjiang[1,2], Ali Khiat[2], Iulia Salaoru[2], Christos Papavassiliou[3] and Themistoklis Prodromakis[2,3*]

[1]College of Electronic Science and Engineering, National University of Defense Technology, Changsha 410073, P. R. China

[2]Department of Electronics and Computer Science, University of Southampton, Southampton SO17 1BJ, UK

[3]Department of Electrical and Electronic Engineering, Imperial College London, London SW7 2AZ, UK.

[*]Email: t.prodromakis@soton.ac.uk

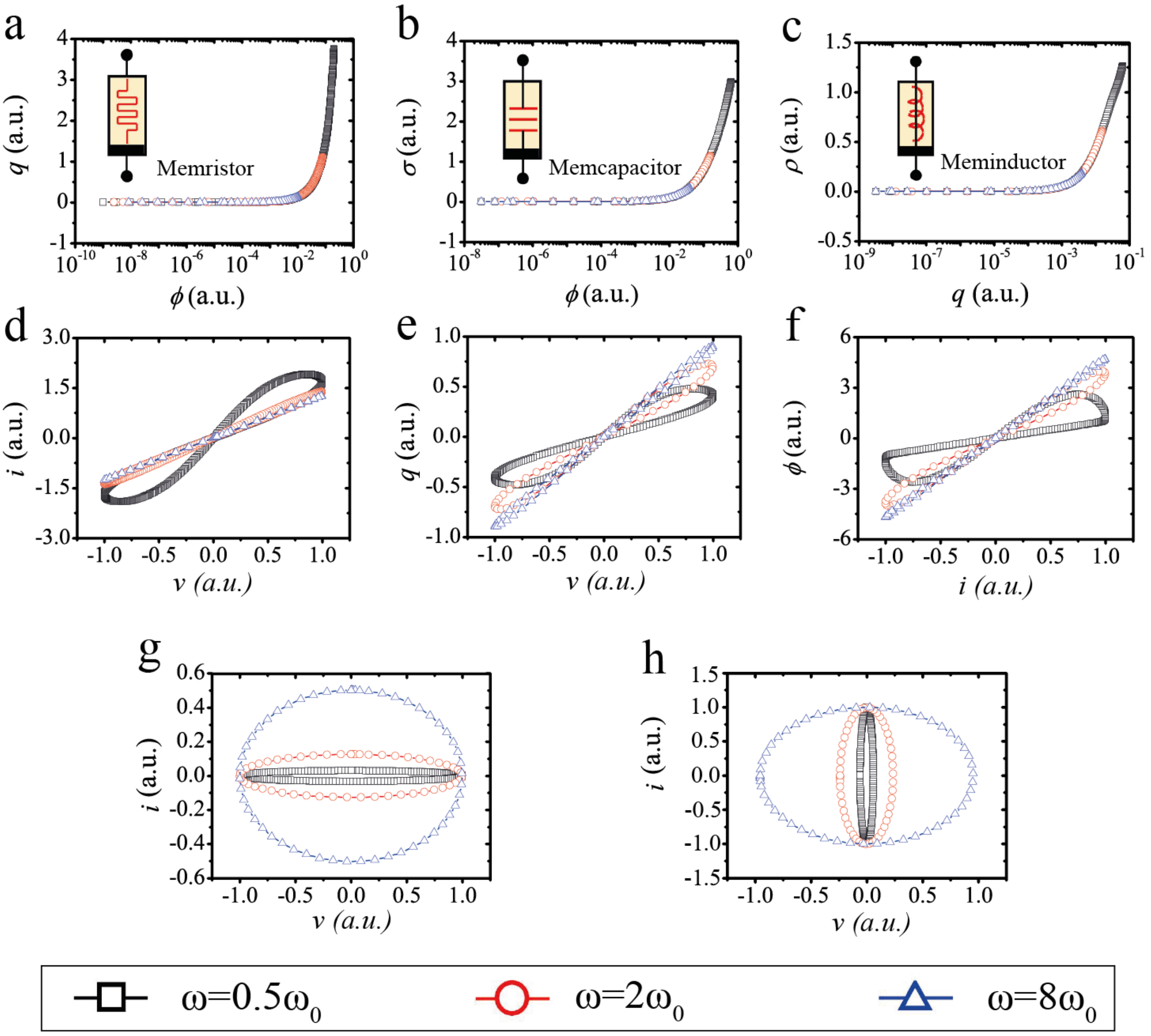

Figure S1 | Theoretical characteristics of memory elements at distinct frequencies. (**a**), (**b**) and (**c**) Definition curves (*q*-ϕ, *σ*-ϕ and *ρ*-*q*) of three memory elements, where $\sigma = \int_{-\infty}^{t} q(t)dt$ and $\rho = \int_{-\infty}^{t} \phi(t)dt$. Insert: Symbols of three memory circuit elements, namely memristor, memcapacitor and meminductor. (**d**), (**e**) and (**f**) Signatures of three memory elements are pinched hysteresis loops in *i*-*v*, *q*-*v* and $\phi - i$ domains respectively. (**g**) and (**h**) *i*-*v* characteristics of memcapacitor and meminductor. Compared to *i*-*v* characteristics of memristor in (**d**), *i*-*v* curves of memcapacitor and meminductor would not cross the origin, while loop areas keep synchronous with improvement of frequency.

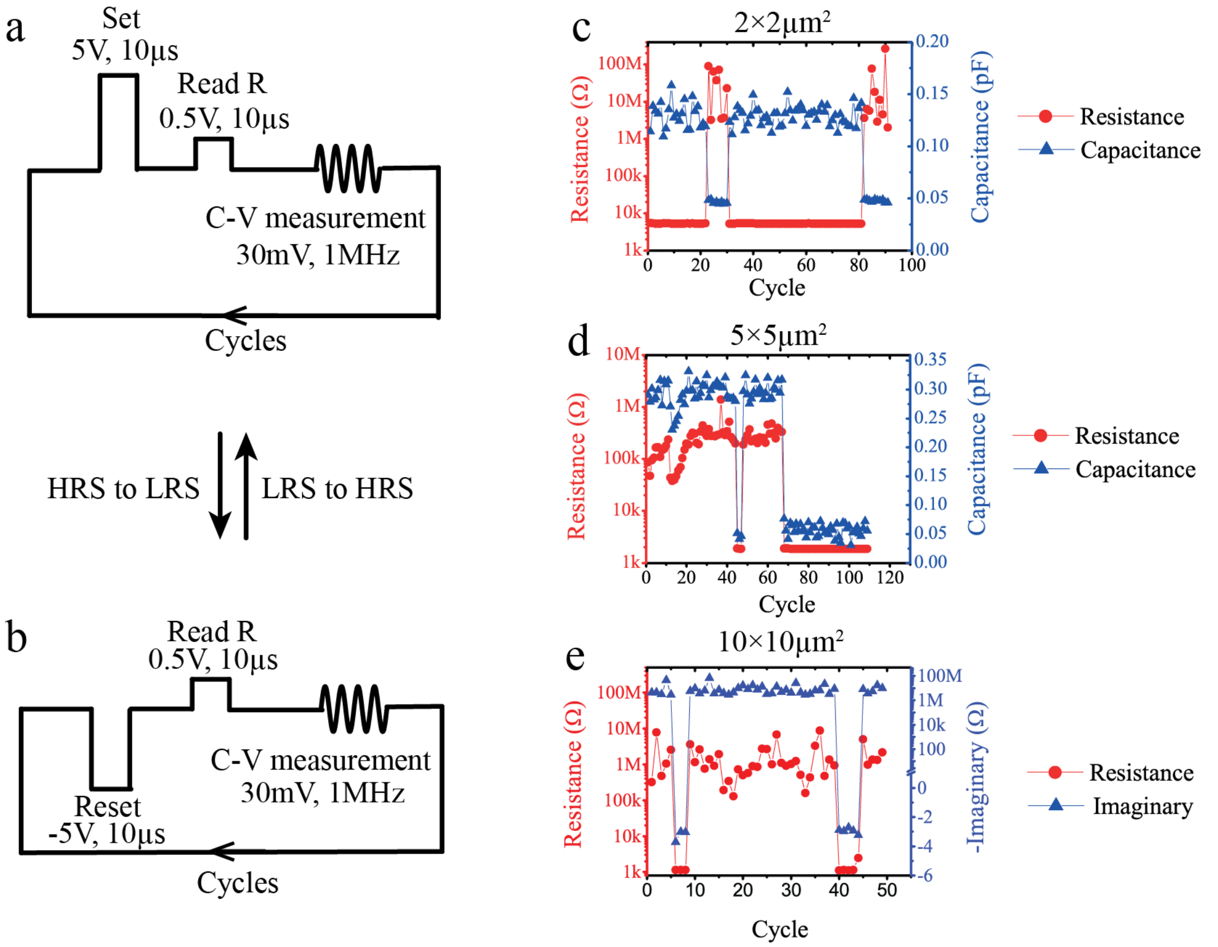

Figure S2 | Impedance evaluating procedure and results on active cells with distinct dimensions. (**a**) Set programming and impedance measurements. After a set pulse, it is followed with resistance reading pulse, C-V measurement at 1MHz. For C-V test, based on the polarity of imaginary part, capacitance values (imaginary is negative) and inductance values (imaginary is positive) would be calculated respectively. (**b**) Reset programming and impedance measurements. It should be noted that for bipolar resistive switching, Set programming measurement cycles would be repeated until resistive states toggled from HRS to LRS, and then Reset programming measurement cycles were implemented until resistance from LRS to HRS. (**c**) and (**d**) Resistance and capacitance changes in repeated measurement cycles on cells with active area of 2×2µm^2 and 5×5µm^2 respectively. It is clear that the changing trends of resistance and capacitance are opposite on devices comprising 2×2µm^2 active cells, while keep synchronous on 5×5µm^2 active cells. (**e**) Programming trends for resistance and imaginary parts of impedance on a 10×10µm^2 active cell. The changes trends of imaginary impedance keep synchronous with that of resistance. However, due to polarities of imaginary values, capacitive effects and inductive effects are dominant alternatively.

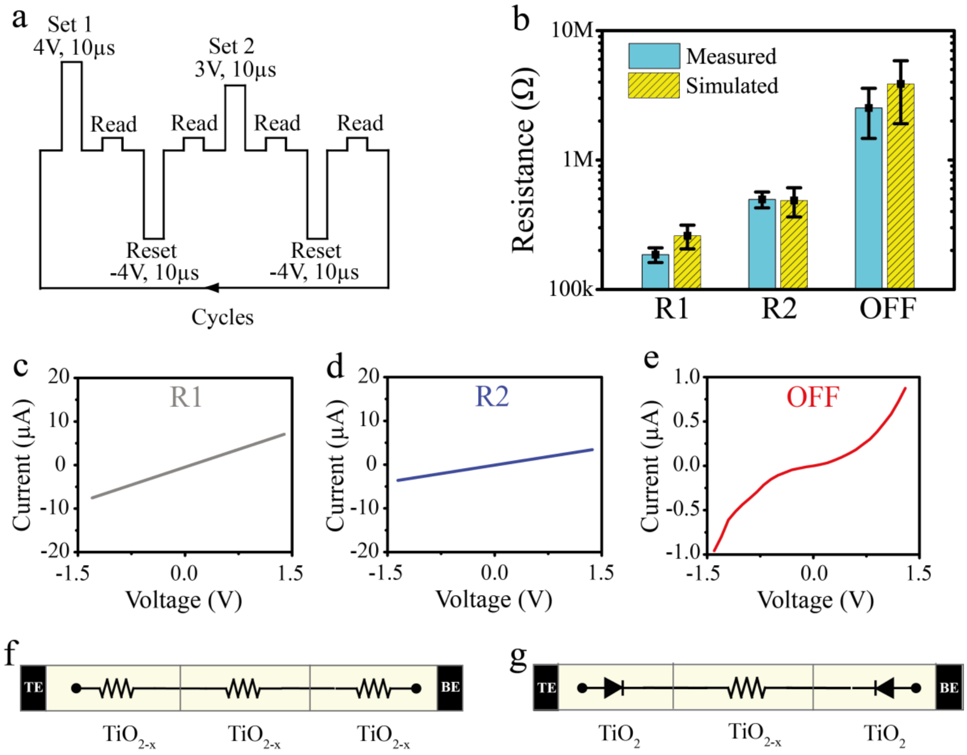

Figure S3 | Evidence of filamentary functional mechanism in MIM ReRAM devices. (**a**) Pulse-induced programming scheme. Each resistance state was obtained with a 0.5V, 1ms pulse, which would not disturb resistance states. (**b**) Three measured (blue) and simulated (yellow) resistive states (OFF, R1 and R2). Three measured states were obtained after employing distinct programming potentials as shown in (**a**). Simulated resistance states were emulated by employing Random Circuit Breaker (RCB) network model with distinct percolation channels and distribution of percolation branches added in the network. (**c**) and (**d**) *i-v* characteristics of ReRAM cell when programmed at R1 and R2 resistance states indicating an Ohmic response. (**e**) *i-v* characteristic of ReRAM cell when programmed at OFF resistance state indicating a rectifying response. Equivalent circuits that denote the effect of the electrodes' interfaces and bulk core are respectively shown for R1 and R2 states in (**f**) and the OFF state in (**g**).

Fig. S3b illustrates three measured resistive states (for resistance, OFF>R2>R1) that were obtained from 50 repeated pulsing sequences with distinct programming potentials (detailed programming scheme was given in Fig. S3a). Then, relative small ±1.5V sweeping potentials were applied at each resistive state without provoking any resistive switching. Fig. S3c, S3d and S3e depict corresponding *i-v* characteristics for the above three resistive states. It is worth to note that at OFF state, a rectifying signature was demonstrated in Fig. S3e indicating the existence of Schottky contacts between the electrodes and TiO_2 bulk (schematic of devices was illustrated in Fig. S3g). In contrast, when ReRAM cell was programmed at LRS (R1 and R2), conductive filaments would shunt the Schottky barrier and result in Ohmic *i-v* signatures in Fig. S3c and S3d (schematic of devices was illustrated in Fig. S3f).

To evaluate this phenomenon, a random circuit breaker network model (RCB) was employed. Parameters utilized in the network are set as follows: network dimension 10×10, bulk resistance (R_{OFF}) 10MΩ, and filamentary resistance (R_{ON}) 60KΩ. Considering SET potential would facilitate the formation of conductive filaments, distinct number of percolation channels and distribution of branched were added in the network, with corresponding simulated results shown in Fig. S3b. Specifically, no percolation channel was added in OFF state, while one and three percolation channels were added within the network to evaluate R2 and R1 respectively. The density of percolation branches obey a normal distribution N(70,12), while these defects were evenly distributed. Comparisons between measured and simulated results in Fig. S3b clearly show that the experimental and simulated results are correlated and, are therefore verifying the notion that resistance modulation results from the formation and rupture of conductive filaments.

For each memory device in the equivalent circuit model in Fig. 4g, detailed state equations are given as follows:

Memristor:

$$R_{MEM} = R_{OFF} - \left(R_{OFF} - R_{ON}\right)x \tag{1}$$

$$dx / dt = ki(t) f(x), k = (\mu_v R_{ON}) / D^2 \tag{2}$$

$$f(x) = \left\{1 - ((0.5 - x)^2 + 0.75)^{10}\right\} \tag{3}$$

Memcapacitor:

$$D_{MEM} = D_{ON} + \left(D_{OFF} - D_{ON}\right)x, D_{MEM} = 1 / C_{MEM} \tag{4}$$

$$\frac{dx}{dt} = ki\left(t\right) f\left(x\right) \tag{5}$$

$$f(x) = \left\{1 - ((0.5 - x)^2 + 0.75)^{10}\right\} \tag{6}$$

Meminductor:

$$L_{MEM} = \left[\sqrt{L_{ON}} + x\left(\sqrt{L_{OFF}} - \sqrt{L_{ON}}\right)\right]^2 \tag{7}$$

$$\frac{dx}{dt} = ki\left(t\right) f\left(x\right) \tag{8}$$

$$f(x) = 10 * \left\{1 - ((0.5 - x)^2 + 0.75)^{10}\right\} \tag{9}$$

Table S1 | Simulation Parameters utilized in equivalent circuit model

Stimulus	Magnitude: 6V, Frequency: [1,10MHz]			
	Switching range	Initial state	Ion mobility	Window Function
Memristor	R_{OFF}=200KΩ<br>R_{ON}=2 KΩ	R_{INI}=200KΩ<br>D=10nm	μ_v=1e-14	$f(x)=(1-((0.5-x)^2+0.75)^{10})$
Memcapacitor	C_{OFF}=3pF<br>C_{ON}=0.1pF	C_{INI}=3pF	k=1e7	$f(x)= (1-((0.5-x)^2+0.75)^{10})$
Meminductor	L_{OFF}=7 µH<br>L_{ON}=0.35 µH	L_{INI}=0.35µH	k=10	$f(x)=10*(1-((0.5-x)^2+0.75)^{10})$
Nanobattery	V_{emf}=0.04V			

For simulations of the proposed equivalent circuit model, parameters settings are demonstrated in Table S1. Considering the pulse-induced programming resistive switching could be incomplete, parameters selected for the memcapacitor are a little larger than the measured results in Fig. 2c (0.1pF-0.3pF). However, it should be noted that in repeated simulation cycles, the initial states for three memory elements are not fixed, which is different from Biolek's models. Specifically, when biased with saturation stimulus, the internal variable cannot return exactly the same value with pristine initial state in an individual simulation cycle. Therefore, in this work, simulation will make use of the final state of internal variable in each individual cycle as the updated Initial state of next simulation cycle. By this way, the accuracy of simulation results could be improved.

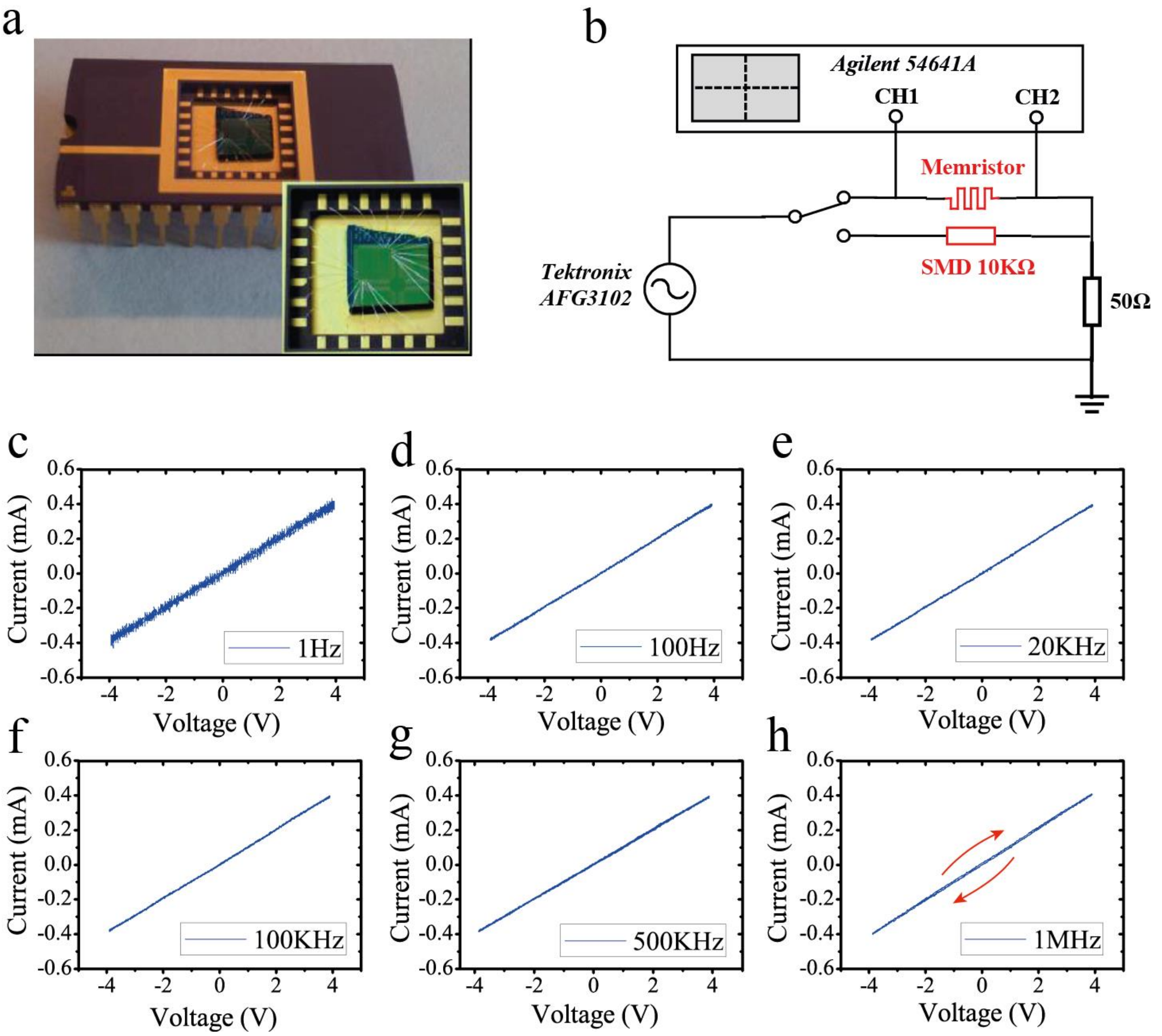

Figure S4 | *I-V* curves measuring circuits and benchmark test employing SMD devices. (**a**) 24-pin package of diced dies. Insert: a close-up of the bonded devices. (**b**) Schematic of *i-v* curves measuring circuits. To distinguish current-voltage characteristics induced by ReRAM cells from influence of cables or other issues, a 10KΩ SMD resistor is employed to implement benchmark test. (**c**) - (**h**) *i-v* curves of comparing benchmark test at frequencies from 1Hz to 1MHz. As (**c**) – (**g**) shows, in our optimised measuring circuit, cables would not introduce significant influence from 1Hz to 500KHz. For measurement at 1MHz in (h), *i-v* curves exhibited capacitive behaviour, which however, is insignificant and completely different from the inductive behaviour in Fig. 4 (f). Therefore, frequency responses in Fig. 4 are definitely from our TiO_2 based ReRAM active cells.

In order to verify the reactance trends in Figs. 2 and 3, we implemented C-V tests in the frequency sweeping mode, and results were depicted as the Bode plots in Figs. S5a-b and Figs. S5c-d, which corresponds to devices with active area of 5×5μm^2 and 10×10μm^2 respectively.

As demonstrated in Figs .S5a and S5b, the frequency dependent results on 5×5μm^2 devices correlate with the demonstrated equivalent circuit structure in Fig. 2, namely a parallel combination of a memristor and a memcapacitor regardless of employed C-V test frequencies. Specifically, within the frequency range [100KHz, 1MHz], both impedance magnitude and phase angel (negative) possessed larger values at the initial state, and then toggled to the lower bonds when the device was switched, which indicates the correlated resistive/capacitive switching. In case of the device with active area of 10×10μm^2, the measured results at the initial state were similar to those on the 5×5μm^2 device, though the magnitude and angle values are slightly different. However, when the device was switched to LRS, the phase angle increased significantly to be positive (500KHz – 1MHz) indicating of the coexistence of capacitive and inductive effects, which verified the reactance trends in Fig. 3b.

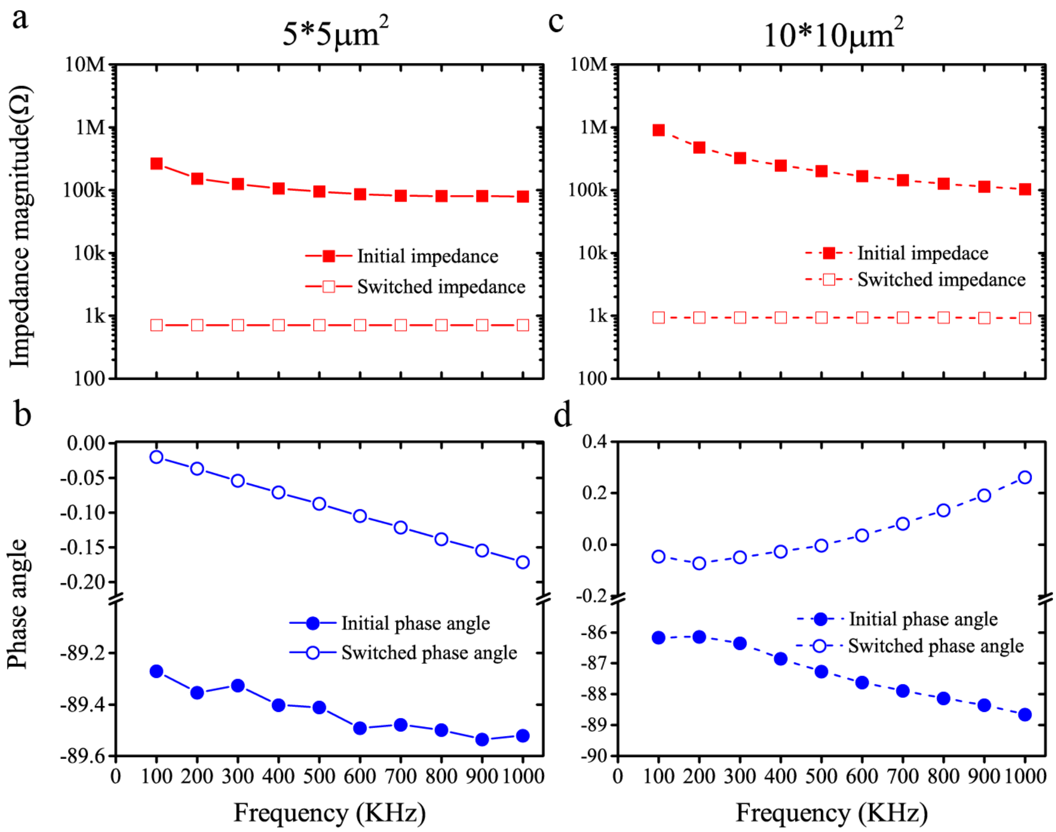

Figure S5 | Bode plots of devices with active area of 5×5 μm^2 and 10×10μm^2. (**a**) and (**b**) Impedance magnitude and phase angle (in units of degree) change versus the frequency of C-V test on a device with active area of 5×5μm^2. (**c**) and (**d**) Impedance magnitude and phase angle (in units of degree) change versus the frequency on a device with active area of 10×10μm^2.

To explore the influence of electrode areas on the measured capacitance, we implemented C-V tests (30mV, 1MHz) on pristine devices before any electrical characteristics measurements to minimise the formation of defects within active cores. The measured initial capacitance of devices with different active areas is shown in Table S2. It should be noted that all tested devices are within the same wafer die and possess the same thickness.

Table S2 | Initial capacitive states of devices with different electrode areas

Electrode areas	Initial capacitance (pF)	Initial capacitance per unit area C_{unit} (pF/μm^2)
2×2μm^2	0.174	0.0435
5×5μm^2	0.46	0.0184
10×10μm^2	1.8	0.0180

As expected, the measured initial capacitance as shown in Table S2 is in proportion to the electrode areas. Then the capacitance per unit electrode area (C_{unit}) was calculated for three distinct device dimensions (2×2μm^2, 5×5μm^2, and 10×10μm^2) respectively. The C_{unit} values of devices with electrode area of 5×5μm^2 and 10×10μm^2 are quite similar, demonstrating the uniform influence of electrode area on measured capacitance. However, the C_{unit} value on 2×2μm^2 cell is relatively larger, indicating possible additional capacitance contributions from the top and/or bottom interfaces on 2×2μm^2 cells as argued in the main text, and also from the disproportionately high fringing field contributions. The capacitance per electrode area could thus be obtained based on experimental data from larger devices (5×5μm^2, and 10×10μm^2), namely: C_{unit}=18 (fF/μm^2).
